# Artificial Intelligence‐Guided Phenotypic Drug Repurposing Against *Streptococcus pneumoniae*

**DOI:** 10.1002/advs.76959

**Published:** 2026-09-16

**Authors:** Joshua S. Fitch, Muhammad D. Ariadi, Min Jung Kwun, Leonie Howells, Saiveth Hernandez‐Hernandez, Nicholas J. Croucher, Pedro J. Ballester

**Affiliations:** ^1^ Imperial College London Department of Bioengineering Sir Michael Uren Hub White City campus London UK; ^2^ MRC Centre for Global Infectious Disease Analysis School of Public Health Sir Michael Uren Hub White City campus London UK; ^3^ Imperial College London Department of Chemistry Molecular Sciences Research Hub White City campus London UK; ^4^ Inserm UMR 1307 CRCI2NA Nantes Université Nantes France; ^5^ Parasites & Microbes programme Wellcome Sanger Institute Wellcome Genome Campus Cambridge UK

**Keywords:** antimicrobial resistance, artificial intelligence, drug resistant, streptococcus pneumoniae

## Abstract

The prevalence of antimicrobial resistance (AMR) within the common bacterial pathogen *Streptococcus pneumoniae* makes it a priority for the development of new antibiotics. While artificial intelligence (AI) has recently boosted phenotype‐based repurposing of drugs for other human pathogens, this remains to be investigated for *S. pneumoniae*. Thus, we leveraged ensembles of transformer, graph, and tree models, each trained on a set of 1849 actives along with 34 503 inactives, to prospectively examine 6747 drugs. Of 11 selected candidate antibiotics, nine were found to strongly reduce in vitro growth of *S. pneumoniae* R6, with IC_50_ values of ≤ 0.4 µg/mL. The most potent drugs, thiostrepton and ceftiofur, had IC_50_ values of 0.0001 µg/mL (60.1 pM) and 0.0004 µg/mL (764 pM), respectively. Thiostrepton remained highly potent even against multidrug‐resistant strains, suggesting it could be effectively deployed to treat common non‐invasive *S. pneumoniae* infection as part of antibiotic stewardship efforts.

## Graphical abstract

Integrated artificial intelligence (AI) ensembles identify highly potent repurposed drugs against *Streptococcus pneumoniae*. Prospective virtual screening of 6747 drugs uncovers nine active candidates, including thiostrepton. This drug exhibits low picomolar potency and bypasses established resistance in multidrug‐resistant strains, demonstrating an effective AI‐guided workflow to combat the global antimicrobial resistance crisis.

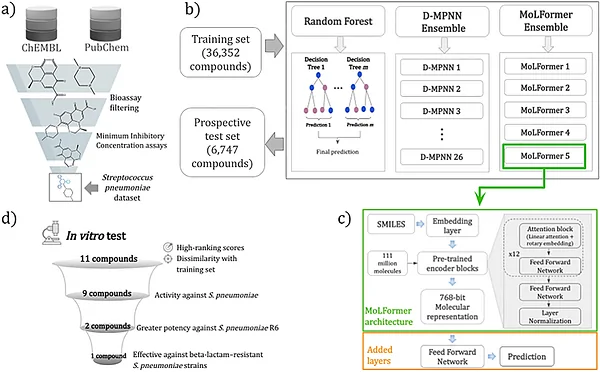

## 1 Introduction

Healthcare costs due to antimicrobial resistance (AMR) could reach $1.2 trillion annually by 2050 [1]. In this context, bacterial AMR is estimated to have caused 1.27 million deaths globally in 2019, with lower respiratory tract infections contributing to this total more than any other syndrome [2]. Treatments effective against drug‐resistant respiratory pathogens, such as *Streptococcus pneumoniae* (the pneumococcus), are therefore urgently needed. In addition to being the most common cause of bacterial community‐acquired pneumonia [3], *S. pneumoniae* is a leading cause of both common infections that drive a substantial proportion of community antibiotic prescribing (e.g. otitis media and pneumonia) as well as more severe invasive infections (e.g. bacteraemia and meningitis) [4]. Pneumococci have become resistant to multiple antibiotics via both the acquisition of resistance genes and the modification of core genes encoding antibiotic targets [5]. Hence, *S. pneumoniae* is one of the twelve bacterial species prioritized by the World Health Organization (WHO) as a target for effective antibiotic development [6].

Computational drug repurposing offers a time‐ and cost‐efficient route to identifying such new treatments for a disease by predicting the effectiveness of drugs with known safety profiles [7]. The most common repurposing approaches have been target‐based, in which it is assumed that effectiveness at treating a disease can be predicted from the inferred ability of a drug to modulate the activity of a macromolecular target. Most of these computational models leverage X‐ray crystal structures of the target [8, 9, 10]. Others model the chemical structures of training molecules [11, 12] and their measured activities for the target to predict the activities of drugs, including those models where the protein sequences of targets are also utilized during training [13, 14]. A drug can also be predicted to have activity for a target if similar molecules have confirmed activities for that target [15, 16, 17]. However, a limitation of all these target‐based drug repurposing approaches is the assumption that potent target inhibition translates to potent whole‐cell activity, which often does not hold [18, 19].

An alternative strategy to drug repurposing is phenotypic screening, which evaluates compounds based on their ability to modulate a biological phenotype, such as cell viability or bacterial growth inhibition, without requiring prior knowledge of their molecular targets. This complementary approach accounts for biological systems operating as complex, interconnected networks, where diseases often arise from system‐wide dysregulation rather than isolated defects in a single protein [20]. By capturing the integrated response of the entire system, phenotypic screening can identify compounds with polypharmacological effects as well as those acting on previously unexplored pathways, which may be critical to achieve therapeutic efficacy. Consequently, phenotypic screening has outperformed target‐based approaches, yielding almost twice as many first‐in‐class small molecule drugs between 1999 and 2008 [21].

Recently, drug discovery and repurposing studies have increasingly used artificial intelligence (AI) methods [22, 23, 24]. While challenging to apply effectively [25, 26], AI has already been successfully applied to drug repurposing [27] in general and to antibiotic discovery [28] in particular. Physicochemical descriptors and chemical structure fingerprints are used as features for well‐established learning algorithms such as Naive Bayes, Support Vector Machine, and Random Forest [29, 30]. More recently, a graph deep learning algorithm called directed message‐passing neural network (D‐MPNN) has been used to predict which existing drugs could be repurposed for use against pathogens in the WHO priority list (e.g., for *Escherichia coli* [31, 32], *Acinetobacter baumannii* [33]). This AI‐guided drug repositioning approach has also been applied to *Staphylococcus aureus* using a multi‐task XGBoost algorithm instead [34]. Overall, these AI models managed to identify drugs with MICs in the range 2–8 µg/mL against these pathogens.

Such proof‐of‐concept studies suggest that AI‐guided repurposing of drugs could also be an important source of new treatments with manageable toxicity for other pathogens such as *S. pneumoniae*. Additionally, improved learning approaches are continuously being identified for such studies (e.g., ensembles of D‐MPNN models performing better than a single D‐MPNN model [35]). Furthermore, specialized pre‐trained models are now available, such as MoLFormer [36], a task‐agnostic transformer‐based model pre‐trained on the SMILES strings of 111 million unlabeled molecules. Given its superior retrospective performance over even D‐MPNN [36], MoLFormer constitutes a promising and yet‐to‐be‐explored approach to phenotypic drug repurposing for antibiotics.

Thus, we curated a dataset and used it to train ensembles of three diverse learning algorithms: Classification and Regression Trees (CART), representing tree algorithms and ensembled as a Random Forest (RF); D‐MPNN ensembles, representing graph neural networks; and MoLFormer ensembles, representing pre‐trained transformer neural networks. These models were then used together to screen a drug library prospectively for promising repurposing opportunities. Finally, the top‐ranked drugs underwent in vitro validation to assess their antibacterial potency, structural novelty, and strain‐specific resistance profiles.

## 2 Materials and Methods

### 2.1 Dataset Preparation

**FIGURE 1:**
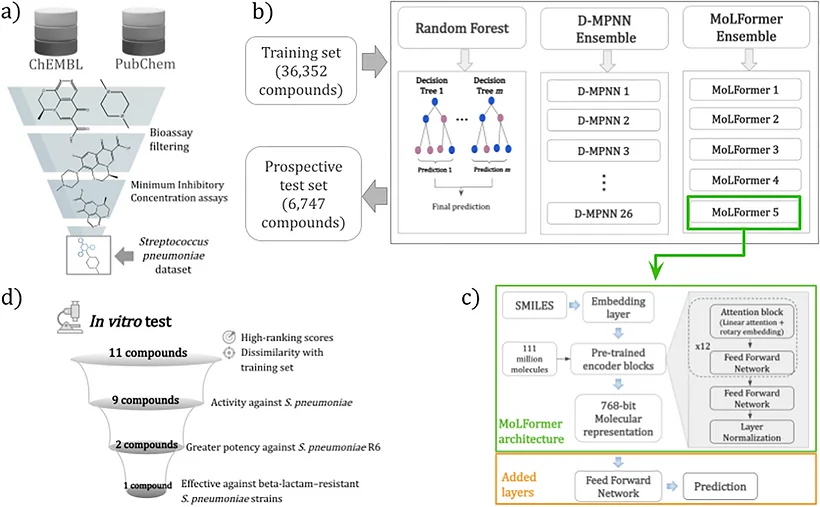
Overview of the AI‐driven drug repurposing workflow against *Streptococcus pneumoniae* cultures. (a) The dataset was created from bioassays from PubChem and ChEMBL, filtered specifically by keywords related to pneumococci and resistance, and further refined to include only bioassays with specific assay types such as MIC, MIC_90_, MIC_50_, and Activity. (b) This study used three AI models to prospectively evaluate 6747 small‐molecule drugs for their potential to inhibit *S. pneumoniae*, selecting those with high consensus predictions as promising repurposing candidates. (c) MoLFormer models are adapted for antibiotic screening by adding a trainable Feed‐Forward Network (FFN) block (highlighted in orange). (d) From the top predictions, we selected 11 compounds for in vitro testing based on high model scores and chemical structure dissimilarity to training molecules.

The dataset was compiled from bioassays retrieved from PubChem and ChEMBL, filtered using the following keywords in the assay titles: “Streptococcus pneumoniae”, “pneumococcus”, or “pneumococci”, combined with “resistant”, “pneumococci EryRc”, or “pneumococci PenR”. The dataset was further refined to only include bioassays with assay type “MIC”, “MIC90”, “MIC50”, and “Activity” (Figure 1a). Most bioassays included in the dataset were conducted with erythromycin‐resistant strains such as A22072, AB11, and B1 (29%) and various penicillin‐resistant strains (29%).

Minimum Inhibitory Concentration (MIC) assays measure the minimum concentration of compounds required to inhibit bacterial growth in an in vitro assay. MIC_90_ and MIC_50_ are variants defined as the minimum concentration of compounds required to inhibit the growth of 90% or 50% of isolates [37]. Half‐maximal inhibitory concentration (IC_50_) is the concentration to inhibit growth of a single isolate by 50% compared to an untreated control. Entries in non‐molar units (e.g., µg/mL) were converted to µM. As 10 µM is commonly taken as a reasonable cutoff for antibiotic activity in antibiotic development [38], industry high‐throughput screening (HTS) [39] and phenotypic anticancer screens [40], values of the MIC, MIC_90_, or MIC_50_ above 10 µM were labeled as negative instances while MIC or MIC_90_ ≤ 10 µM and MIC_50_ ≤ 5 µM were labeled as positive instances. We use a 10 µM cut‐off for MIC_90_ as this means it is very likely an individual isolate has MIC at least this low (usually lower), and a more stringent cut‐off of 5 µM for MIC_50_, as it is easier to inhibit 50% of a population than 90%. Merging measurement types has been successful for similar work in the past [31], and this conservative conversion reduces the chance of false positives whilst allowing us to enlarge the training set by integrating slightly different label types. Sometimes a molecule has multiple entries because it has been tested in vitro several times, with potentially different measurement types. If all labels of the multiple entries of a molecule belong to the same class, then just one of the multiple entries was retained. By contrast, if there was ambiguity in the label of a molecule (i.e., at least one entry in multiple classes), then that molecule was discarded. In this way, only molecules that clearly belonged to one of the classes were used in the test set, thus ensuring ground truth clarity.

Optimizing the model for screening large compound libraries, typically characterized by diverse chemical structures and a small percentage of active molecules [41, 42], benefits from supplementing the training set with a varied set of inactive molecules. This occurs because positive outcomes tend to be reported and published more frequently than negative outcomes, meaning datasets curated from public sources are more likely to contain a large proportion of active compounds, thereby biasing the training of these models. Thus, to better reflect the characteristics of large compound libraries, we augmented the compiled dataset with 33 488 molecules that were found to induce less than 50% growth inhibition at a drug concentration of 50 µM in an HTS for another drug‐resistant gram‐positive bacterium, methicillin‐resistant *S. aureus* (MRSA) [41]. Assuming that molecules inactive for MRSA are also inactive for *S. pneumoniae* is an accurate modeling choice for multiple reasons. First, molecules selected for HTS are mostly inactive for a given pathogen, e.g., a low hit rate (0.6%) in a *S. pneumoniae* HTS screen despite using a weak activity threshold [43]. Second, type strains of *S. aureus* and *S. pneumoniae* respond to antibiotics similarly. For example, in the BacDive database [44], there are 47 antibiotic‐concentration pairs tested for disk diffusion inhibition diameter on both *S. pneumoniae* and *S. aureus*, and the type strains for each species have a high correlation of 0.80 (p = 1.7e‐11), making these HTS molecules even less likely to be active for *S. pneumoniae*. Third, several studies have also used assumed inactives (just assumed, not even inactive for a related target as us here) and found that such inactive‐enriched training sets improved hit rates in target‐based virtual screening [45]. If assumed inactives in the training set were actually active, then these label errors would degrade model performance and hit rates would not have improved.

To categorise this dataset into groups of similar structures [46], the molecules were compared using their 2048‐bit Morgan fingerprints (radius 3). 4812 outlier molecules (98% of which were MRSA negatives) with Tanimoto similarity < 0.5 to any other molecule were removed to avoid skewing the clustering algorithm [47]. The final composition of the dataset is shown in Table 1.

**TABLE 1:** Overview of the compiled dataset used to evaluate AI‐driven phenotypic models for drug repurposing against *S. pneumoniae*. Data were retrieved from PubChem and ChEMBL. The final dataset includes compounds with known activity against *S. pneumoniae* (positive class) and decoy compounds tested initially against MRSA (negative class). Compounds are labeled as active (positive class) if their MIC value is ≤10 µM, and inactive (negative class) otherwise. The number of unique compounds per class is reported for each assay type. The total number of unique compounds across all bioassays is also provided.

Label	Assay type					Total
	MIC	MIC_90_	MIC_50_	Activity	MRSA activity	
Positive	1820	12	4	13	0	1849
Negative	1004	2	2	7	33 488	34 503
**Total**	2824	14	6	20	33 488	36 352

The similarities of remaining molecules were summarized as a two‐dimensional UMAP representation using the Jaccard distance, 100 neighbors, and 0 as the minimum distance. Subsequently, agglomerative hierarchical clustering based on the Euclidean distance was used to cluster the UMAP representation into a pre‐determined number (K) of clusters. The quality of the resulting cluster is measured through the Silhouette score (high quality corresponds to high similarity of molecules within each cluster relative to the similarity observed between clusters). The Silhouette scores were calculated for a range of K from 3 to 50 and the K value with the highest Silhouette score (K = 26) was taken as the optimum number of clusters. The composition of clusters can be seen in Table S1.

This cluster‐based splitting was used when developing deep learning models to create varied and robust ensembles trained on distinct parts of chemical space, whereas simpler stratified splits were used when developing the classical RF model. This enabled the generation of a diverse set of statistical models that should complement one another in the virtual screen. All models were trained as binary classifiers.

### 2.2 Training RF

We chose a simple and standard procedure for RF generation to identify the most easily detectable similarities between the training set and candidate compounds for testing. We trained RF using radius‐3, 2048‐bit Morgan fingerprints as molecular representations, implemented in scikit‐learn [48]. Hyperparameters were optimized via grid search with fivefold stratified cross‐validation on the dataset. Ranges were picked for some of the most influential hyperparameters (maximum depth, minimum samples per leaf, criterion, and balance) based on commonly used values in the literature [49]. Tuning maximized area under the precision‐recall curve (PR‐AUC), a commonly used metric in virtual screening where datasets are imbalanced [50], the tuning grid can be seen in Table S2. The best‐performing configuration was then retrained on the full dataset (Figure 1b).

### 2.3 Training D‐MPNN Ensemble

A D‐MPNN ensemble of 26 models was developed. D‐MPNN adopts the Chemprop graph neural network architecture [35]. Each D‐MPNN model was trained with a different UMAP cluster held out to increase ensemble diversity, and the final output was taken as the median prediction across models. Since the Chemprop defaults have already been tuned for molecular property prediction, we employed them rather than performing a new hyperparameter search for such a large ensemble. Thus, hyperparameters were left at their default values, with three exceptions: we increased the capacity of the feed‐forward network, introduced some dropout to mitigate overfitting, and replaced ReLU with GELU activations, which have been shown to improve stability in deep architectures [51]. Additional training details for D‐MPNN can be found in Table S4.

### 2.4 Training MoLFormer Ensemble

MoLFormer [36] builds upon the standard transformer encoder architecture introduced for natural language processing [52]. MoLFormer is pre‐trained with a masked language modeling task on 111 million SMILES strings of molecules from the PubChem and ZINC databases. This pre‐training aims to learn information‐rich universal molecular representations that capture higher‐order features, for example relating to structure or molecular properties. These representations have been adapted to achieve state‐of‐the‐art performance on many molecular property prediction benchmarks [36].

We adapted MoLFormer models for antibiotic screening in two ways. First, by adding feed‐forward layers on top of the frozen pre‐trained transformer (Figure 1c) to predict the probability of activity against *S. pneumoniae*. Second, by generating an ensemble, in which each component MoLFormer was trained with a different UMAP cluster held out to increase diversity. Our ensemble comprised 5 of these transformer‐based molecular language models, each of which was trained using data from all clusters but the held‐out cluster that had the most similar proportion of positives to the overall dataset, as seen in Table S1. This ensured all models in the ensemble were appropriately calibrated for application to screening large molecular datasets. Predictions were aggregated by taking the median across the ensemble.

To efficiently fit the models whilst still tuning on dissimilar molecules, hyperparameters were tuned on a single MoLFormer model with cluster 0 (the remaining cluster with the most similar positive proportion to the overall dataset) as the validation set and then copied for all models in the ensemble. A small set of sensible ranges for important hyperparameters (hidden dimension, number of layers, dropout) was selected and tuned using the Ray Tune library in grid search mode, with unpromising trials that had low PR‐AUC during the first few epochs stopped early. This procedure balanced finding the best hyperparameters with computational time. The tuning grid can be seen in Table S3, and additional training details for MoLFormer can be seen in Table S5. To our knowledge, this work represents the first application of a pre‐trained molecular language model in an antibiotic screening setting.

### 2.5 Microbiological Experiments

Compounds were dissolved in water or dimethylsulphoxide, depending on their solubility. Each growth assay used an inoculum of 2 × 10^4^ *S. pneumoniae* R6 cells from titrated frozen stocks, stored in 10% glycerol. These bacteria were then cultured in an individual well of a 96‐well microtiter plate in a 5% CO_2_ atmosphere at 35°C, growing in 198 µl of a 2:3 ratio mixture of Todd‐Hewitt broth (Sigma–Aldrich) with 0.5% yeast extract (Sigma–Aldrich), and Brain‐Heart Infusion media (Sigma–Aldrich), supplemented with 2 µl of the solution of the compound being tested. Cell density was quantified as the optical density at a wavelength of 600 nm (OD_600_), measured at 30 min intervals using a FLUOstar Omega microplate reader (BMG LABTECH). Three independent biological replicates were grown for each concentration of each tested compound.

## 3 Results and Discussions

### 3.1 AI‐guided Repurposing of Drugs

The diverse AI models (RF, D‐MPNN ensemble, and MolFormer ensemble) were independently trained on the experimentally‐characterized dataset of 36 352 molecules (see Methods), to maximize the chances of the virtual screen identifying high‐confidence predictions of molecules likely to be active against *S. pneumoniae*. Each was then used to rank the molecules in the Drug Repurposing Hub (March 24^th^ 2020 release), a separate library of 6776 approved or investigational drugs [53] based on their positive class probabilities (i.e. their predicted likelihood that the drug inhibits the growth of *S. pneumoniae*), which corresponded to the median of the constituent model predictions for the ensemble methods. After removing any drug that was also tested on *S. pneumoniae* in the training set, the outputs of the models were compared using their rankings of the remaining 6747 drugs.

**FIGURE 2:**
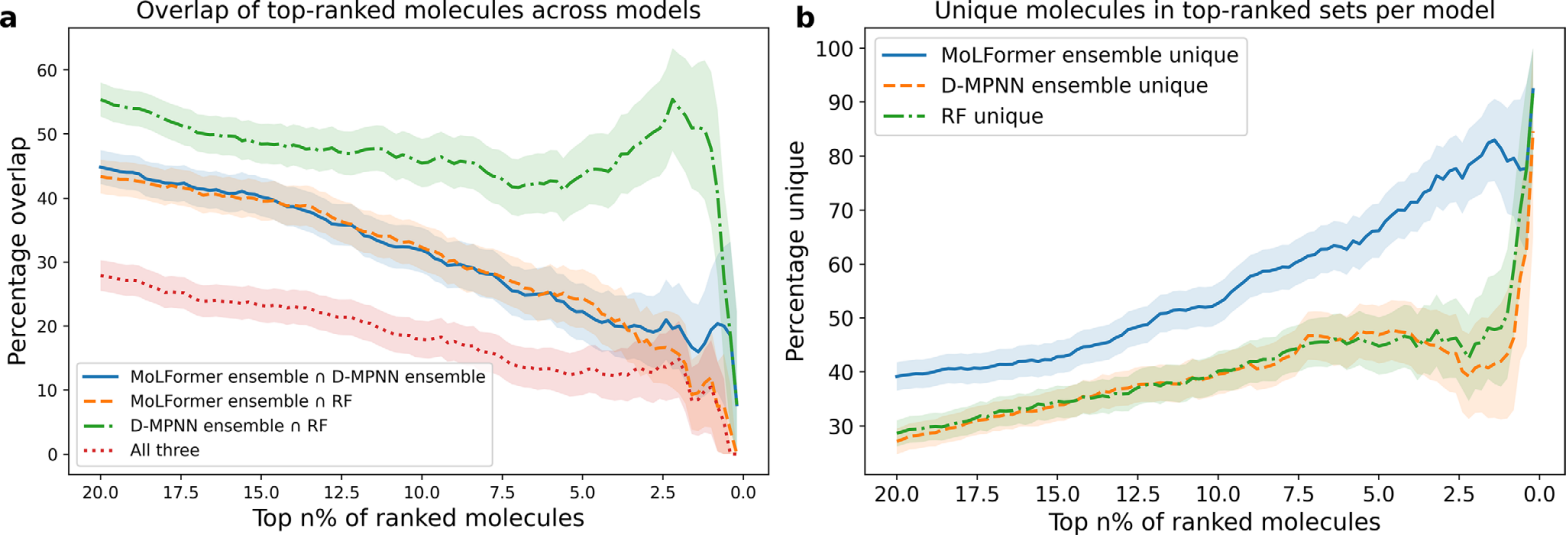
Analysis of the diversity of different AI model outputs. (a) Percentage overlap of molecules in the top‐n‐quantile of ranks for each model. Shaded regions are 95% confidence interval calculated with a normal approximation for a binomial proportion. (b) Plot of the proportion of molecules unique to a model, defined as the fraction of molecules in the top n% of a model that do not appear in the top n% of any other model. Shaded regions were calculated the same way as in panel (a).

To determine whether both deep learning methods had converged on predictions that were distinct from the RF outputs, we analyzed the differences in rankings between the models (Figure 2a). Unexpectedly, the RF and D‐MPNN ensemble had the highest overlap and were more than twice as similar as either model was with the MoLFormer ensemble when considering the highest 1%–5% of rankings. The MoLFormer ensemble had an overlap below 50% at all n threshold values, and below 30% with the D‐MPNN ensemble and RF at higher ranks (n≤8%).

The proportion of molecules unique to each method was also calculated at different quantile thresholds (Figure 2b). These trends followed the same patterns as Figure 2a, emphasizing that the MoLFormer ensemble generated the most divergent model predictions. It retained strong uniqueness in predictions compared to the D‐MPNN ensemble and RF up until n ≤0.5, at which point sparsity resulted in a spike in uniqueness and overlapping standard errors for all models (Figure 2a,b). This suggests the pre‐trained transformer model prioritizes molecular patterns that other models do not pick up on.

Due to the redundancy between D‐MPNN ensemble and RF rankings, we decided to primarily use MoLFormer ensemble and D‐MPNN ensemble when selecting molecules for prospective validation. D‐MPNN was chosen over RF for its higher complexity and success in previous studies [32]. We retained the 19 drugs where the mean of the activity probabilities from the MolFormer ensemble and D‐MPNN ensemble was ≥0.7. To complement this consensus‐based approach and maximise the finding of unique hits, we also considered the top 10 molecules from each individual model (RF, D‐MPNN ensemble, MoLFormer ensemble). This process led to the pre‐selection of 36 unique drugs, which were then manually screened, which allowed the additional important factors listed below to influence selection and find more realistic candidates.

Of this set, 25 drugs could not be inexpensively or easily synthesized or were already known to have *S. pneumoniae* activity, or were very similar to other drugs in the list. This meant they were either difficult to test and less likely to be viable as treatments or redundant. Therefore, we retained the remaining 11 drugs. 9 of these 11 drugs came from the consensus of the two deep learning models, 1 only from RF (levofloxacin), 3 from the D‐MPNN ensemble (thiostrepton, cefozopran, cefotiam‐cilexetil; the first two drugs were also highlighted by the consensus model), and 3 from the MoLFormer ensemble (thiostrepton, dactinomycin, cadazolid; all three drugs were also highlighted by the consensus model). Note that whilst we compare the outputs and ranking of the different ML methods, we make no claims about our procedure being optimal or one ML model being provably superior in this study. Following other similar studies in the literature [54, 55, 56], we designed and focused our study to directly maximize prospective performance. We only compare models with the intent of retrospectively learning from the prospective results.

### 3.2 Efficacy of the Repurposed Drugs on a Drug‐Sensitive *S. pneumoniae* Strain

**FIGURE 3:**
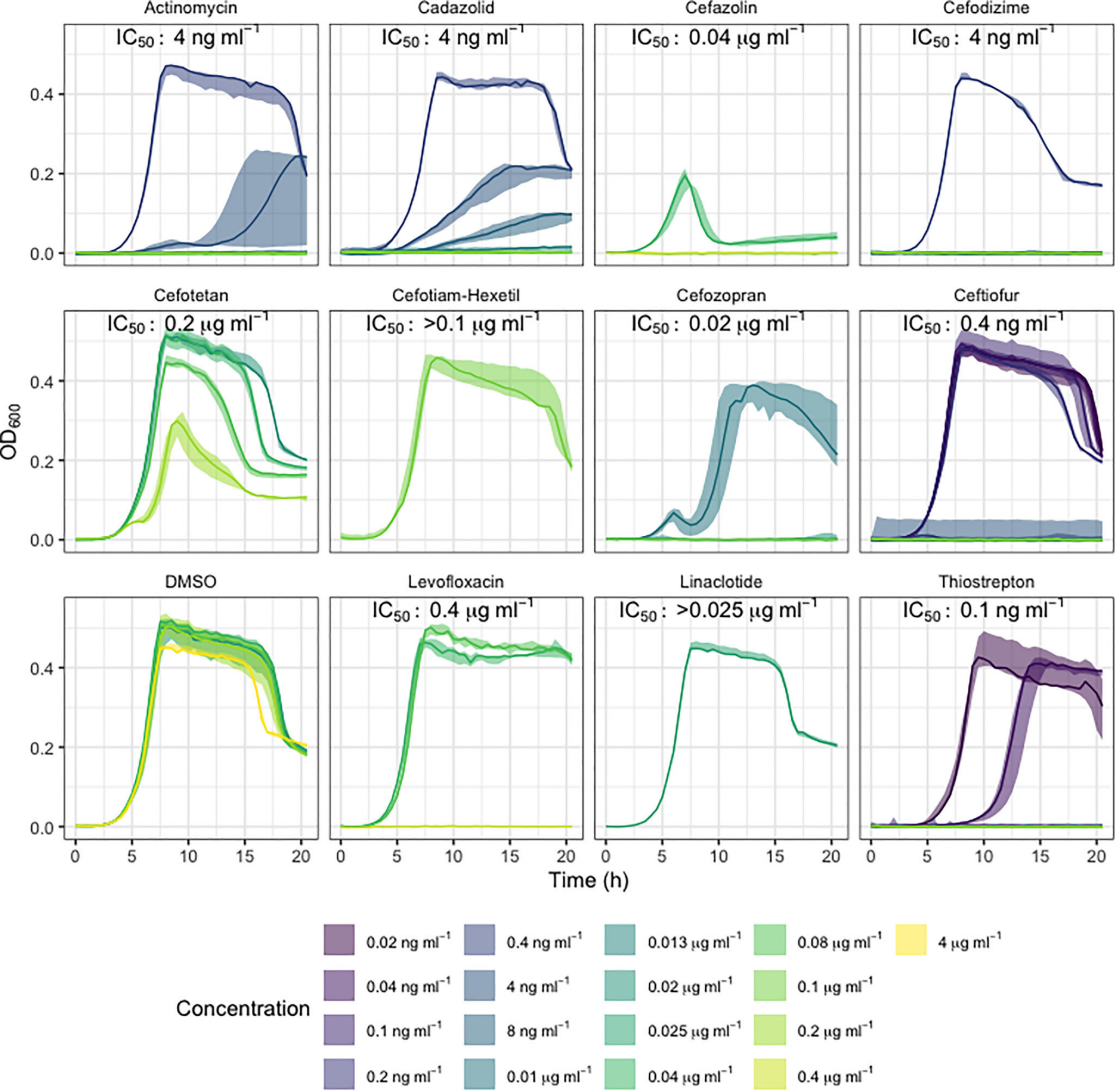
*S. pneumoniae* R6 growth curves showing the effect of each of the 11 candidate antibiotics. Each panel shows the bacterial growth observed for different concentrations of a particular candidate molecule. For each tested candidate antibiotic concentration, corresponding to a defined color, bacterial growth was measured using the optical density at 600 nm (OD_600_) of three replicate wells. The solid line indicates the median OD_600_, and the ribbon shows the range of measurements across replicates at each time point. The colors represent the concentration of the candidate antibiotic with which the media was supplemented. The exception is DMSO, which was included as a control for the solvent in which each molecule was dissolved. The IC_50_ values were calculated as the minimum concentration that halved the growth density over the period of the experiment.

These 11 compounds were tested in vitro for their effect on pneumococcal growth (Figure 1d). Figure 3 shows the *S. pneumoniae* R6 growth curves for each of these drugs predicted to have activity against this human pathogen. Nine of the drugs (82%) demonstrated activity against *S. pneumoniae*, with IC_50_ values (the minimum concentration inhibiting growth by 50%) of ≤ 0.4 µg/mL (Table 2).

**TABLE 2:** Results from the prospective validation of MoLFormer's drug repurposing predictions. The 11 drugs that were prospectively predicted to be active on *S. pneumoniae* and their IC_50_ determinations following in vitro validation. To our knowledge, only levofloxacin was previously known to have *S. pneumoniae* activity. “Similarity to known” is the highest Tanimoto similarity score between the drug and any of the known 1849 active molecules for this pathogen.

Drug	Known mechanism of action	Similarity to known	Quinolone ring	Beta lactam ring	IC_50_ (µg/mL)	IC_50_ (nM)
Dactinomycin	RNA polymerase inhibitor [60]	0.20	No	No	0.004	3.19
Cadazolid	Bacterial ribosome inhibitor [61]	0.55	Yes	No	0.004	6.83
Cefazolin	Bacterial cell wall synthesis inhibitor [62]	0.36	No	Yes	0.04	88.0
Cefodizime	Bacterial cell wall synthesis inhibitor [63]	0.65	No	Yes	0.004	6.81
Cefotetan	Bacterial cell wall synthesis inhibitor [58]	0.26	No	Yes	0.2	347
Cefotiam‐cilexetil	Bacterial cell wall synthesis inhibitor [64]	0.32	No	Yes	>0.1	>130
Cefozopran	Bacterial cell wall synthesis inhibitor [65]	0.46	No	Yes	0.02	38.8
Ceftiofur	Bacterial cell wall synthesis inhibitor [66]	0.63	No	Yes	0.0004	0.764
Levofloxacin	Bacterial DNA gyrase inhibitor [67]	0.76	Yes	No	0.4	1107
Linaclotide	Guanylate cyclase agonist [68]	0.33	No	No	>0.025	>16.4
Thiostrepton	Bacterial ribosome inhibitor [69]	0.23	No	No	0.0001	0.0601

Structurally, six of these drugs were cephalosporins, which have a beta‐lactam scaffold targeting bacterial cell wall synthesis. Despite this structural similarity, differences were observed in the molecules’ effectiveness against the penicillin‐susceptible *S. pneumoniae* R6. One such compound, cefotiam‐cilexetil, had little effect on pneumococcal growth (Figure 3). This false positive inference by the model can be explained by the compound being a prodrug that is activated by esterases [57] that are absent from in vitro culture. The next highest IC_50_ of the beta‐lactams was cefotetan, at 0.2 µg/mL, a drug that has previously been noted as being more effective against gram‐negative bacteria than streptococci [58]. The molecules cefazolin and cefozopran, respectively classed as first‐ and fourth‐generation cephalosporins, exhibited intermediate levels of effectiveness against pneumococci, with IC_50_ values of about 0.03 µg/mL. This is below the European Committee on Antimicrobial Susceptibility Testing (EUCAST) threshold for benzylpenicillin resistance of 0.06 µg/mL [59], consistent with these antibiotics being viable treatment options. However, the third‐generation cephalosporins (cefodizime and ceftiofur) were the most effective against R6, with ceftiofur completely suppressing bacterial growth at concentrations below 1 ng/mL.

Two of the eleven compounds had a quinolone ring. One was levofloxacin, a fluoroquinolone that is particularly active against gram‐positive bacteria [70]. The other was cadazolid, a first‐in‐class quinoxolidinone antibiotic developed for treating the gram‐positive pathogen *Clostridioides difficile* [71]. Both exhibited similar efficacy against pneumococci as some of the cephalosporins.

Three of the compounds lacked either beta‐lactam or quinolone scaffolds. One was linaclotide, a laxative without any known antimicrobial activity [72]. This had no effect on pneumococcal growth at the tested concentrations. However, the cancer chemotherapy dactinomycin had similar effectiveness against pneumococci as the quinolones and the less‐effective cephalosporins. By contrast, the cyclic oligopeptide antimicrobial thiostrepton was highly effective against *S. pneumoniae*, with the lowest IC_50_ of any of the tested compounds (IC_50_ = 0.0001 µg/mL).

**FIGURE 4:**
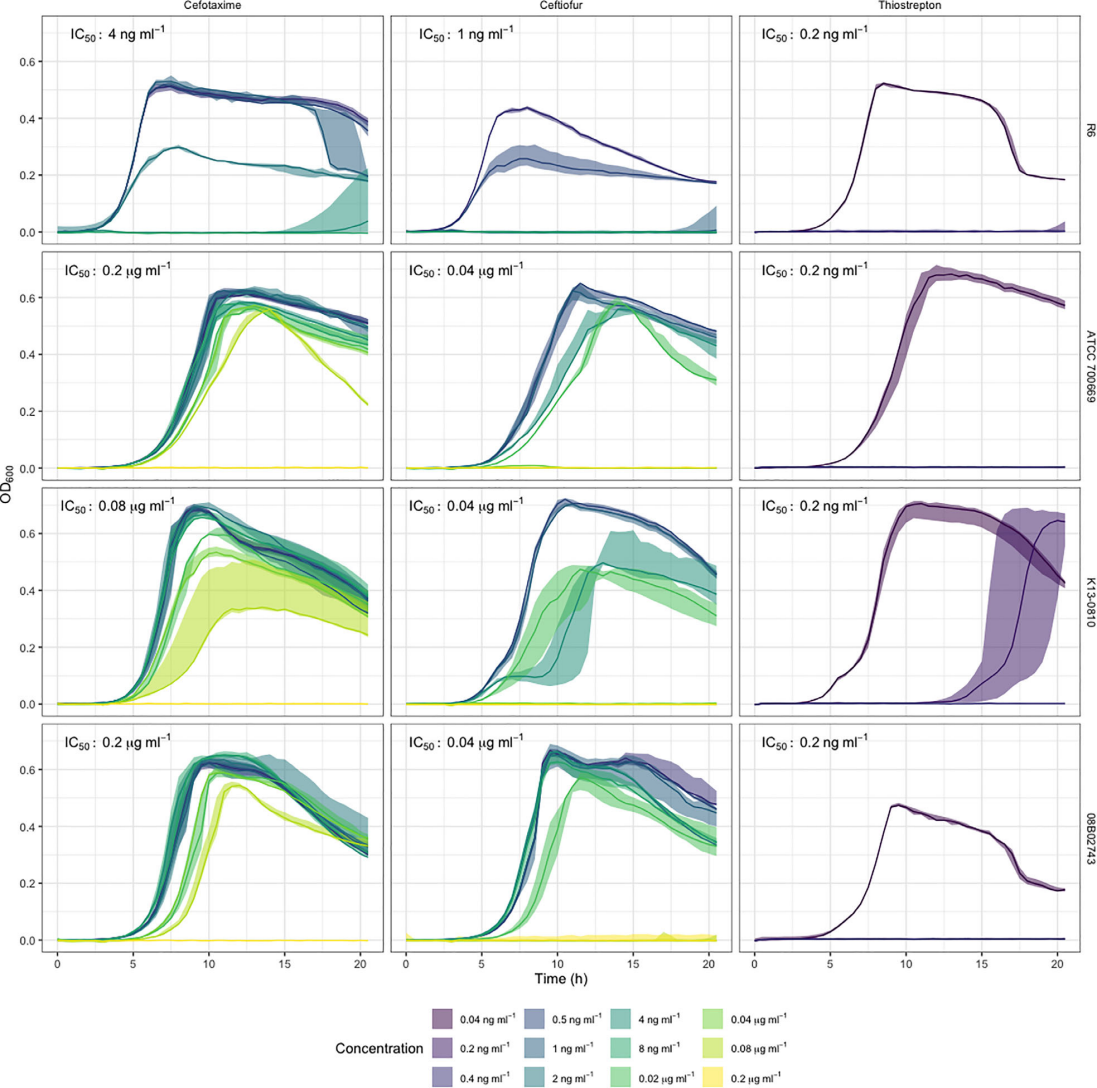
Growth curves of penicillin susceptible and non‐susceptible *S. pneumoniae* for the two most potent repurposed drugs (thiostrepton, ceftiofur). As the positive control, we use cefotaxime, a third generation cephalosporin used very commonly in humans. Line plots showing the growth curves of R6 and penicillin non‐susceptible *S. pneumoniae* in the presence of different concentrations of the cephalosporins cefotaxime and ceftiofur. Data are displayed as described in Figure 3.

As other beta‐lactams are used to treat pneumococcal infections, the effectiveness of ceftiofur was compared with that of the commonly‐used third generation cephalosporin, cefotaxime (Figure 4). This demonstrated that ceftiofur was a more effective drug against R6, as was thiostrepton. Hence, both thiostrepton (a bacterial ribosome inhibitor) and ceftiofur (a third‐generation cephalosporin) exhibited greater potency against *S. pneumoniae* R6 than commonly used antibiotics, with IC_50_ values of just 0.0001 µg/mL (60.1 pM) and 0.0004 µg/mL (764 pM), respectively. The largest similarity to training molecules for each of these 11 drugs is also shown in Table 2. Both drugs are chemically distinct from any molecule in the training set of 1849 actives, with Morgan‐based Tanimoto similarities of 0.23 for thiostrepton and 0.63 for ceftiofur, evidencing that AI models are able to generalize to not only unseen, but also dissimilar molecules.

### 3.3 Evaluating the Most Potent Repurposed Drugs on Drug‐Resistant *S. pneumoniae* Strains

The need for new treatments is most acute for multidrug‐resistant pneumococci, particularly those non‐susceptible to beta lactams and macrolides. As ceftiofur has a beta‐lactam scaffold, and thiostrepton is known to target the bacterial ribosome, the antibiotics were tested against three distinct multidrug‐resistant genotypes: ATCC 700669, a representative of the PMEN1 genotype [73]; K13‐0810, a representative of the PMEN2 genotype [74]; and 08B02743, a representative of the PMEN14 genotype [75]. All three genotypes exhibit reduced penicillin susceptibility due to modifications of their penicillin‐binding proteins, and consequently were more tolerant of both ceftiofur and cefotaxime. Nevertheless, the IC_50_ values of ceftiofur were two‐ to fivefold lower than those of the commonly used cephalosporin cefotaxime (Figure 4). Therefore, the increased potency of ceftiofur relative to cefotaxime was not diminished by changes in the penicillin‐binding proteins.

Each of the three genotypes had a different spectrum of resistance to ribosome‐targeting antibiotics. They all expressed the TetM resistance protein, which protects the ribosome from tetracycline binding; ATCC 700669 also has a chloramphenicol acetyltransferase; K13‐0810 has an ErmB ribosomal methylase, and 08B02743 has a Mef‐type efflux pump. Nevertheless, thiostrepton remained potent against these resistant strains, with IC_50_ values of 0.0002 µg/mL (120 pM). This suggests that thiostrepton has a distinct activity against the ribosome that is not affected by resistance to other antibiotics commonly used to treat pneumococci.

### 3.4 Comparing the Three AI Models Based on Their Prospective Results

The in vitro validation of the compounds selected by the models enabled evaluation of which algorithms were most accurate in identifying highly effective drugs (Table 3). However, this analysis was limited by the selection bias against molecules that scored highly with the RF model. Spearman's rank‐order correlation (ρ) between relative model rank and IC_50_ was calculated and statistical tests were performed (two‐tailed, n = 11). From these, the MoLFormer ensemble (ρ = 0.75, p = 0.0073) and the MoLFormer ensemble‐D‐MPNN ensemble consensus ranking (ρ = 0.74, p = 0.0098) showed significant correlation with potency ranking, whereas D‐MPNN ensemble alone (ρ = 0.15, p = 0.65) and the RF baseline (ρ = 0.16, p = 0.63) do not.

**TABLE 3:** Ranking of the 11 molecules selected for screening by each AI model. Ranks are out of the 6747 drug repurposing hub molecules that are not present in the non‐decoy training set (i.e., the actives). IC_50_ values considered active are in bold. Spearman's rank‐order correlation coefficient (two‐tailed) was used to assess the relationship between model and IC_50_ ranks (n = 11). MolFormer ensemble—D‐MPNN ensemble consensus ranks are based on averaging the median predictions from the MolFormer and D‐MPNN ensembles.

Molecule Name	Consensus of MoLFormer and D‐MPNN ensembles Rank	MoLFormer Ensemble Rank	D‐MPNN Ensemble Rank	Random Forest Rank	IC_50_ (µg/mL)
Thiostrepton	1	1	1	115	**0.0001**
Ceftiofur	8	26	26	38	**0.0004**
Dactinomycin	15	7	145	192	**0.004**
Cefodizime	5	22	11	34	**0.004**
Cadazolid	7	10	46	27	**0.004**
Cefozopran	4	33	4	56	**0.02**
Cefazolin	10	25	28	57	**0.04**
Cefotetan	9	20	34	117	**0.2**
Levofloxacin	38	119	117	7	**0.4**
Linaclotide	18	64	39	168	>0.025
Cefotiam‐cilexetil	97	2525	2	124	>0.1
**Average Rank of Actives**	**10.8**	**29.2**	**45.8**	**71.4**	
**Rank Correlation (Significance)**	**0.74 (0.0098)**	**0.75 (0.0073)**	**0.15 (0.65)**	**0.16 (0.63)**	

Due to the prioritization of MoLFormer ensemble and D‐MPNN ensemble in molecule selection, their p‐values are likely more inflated than that of RF. This inflation and the low sample size (n = 11) mean conclusions made from this analysis are necessarily speculative. With that said, the large difference in rank correlation of the MoLFormer ensemble compared to the D‐MPNN ensemble and RF suggests that the higher‐order features captured by transformer‐based embeddings might generalize better to screening novel molecules. Furthermore, the two drugs selected by the MoLFormer ensemble for in vitro tests (thiostrepton, dactinomycin) are the most dissimilar to any molecule with *S. pneumoniae* activity in the training set (Table 2). Further studies with a focus on prospectively comparing different ML methods are required to see if these trends hold under bigger sample sizes and more uniform training regimes.

Despite the stronger performance of the MoLFormer ensemble, no model would have identified all active compounds by itself. Thiostrepton was ranked first by both the D‐MPNN ensemble and MoLFormer ensemble, but only the 115^th^ by RF. Both the D‐MPNN ensemble and MoLFormer ensemble ranked levofloxacin around the 120th, but RF ranked it seventh. Lastly, both the D‐MPNN ensemble and RF ranked dactinomycin lower than the 140^th^, but the MoLFormer ensemble ranked it seventh. Collectively, this shows how every model misses some of the drugs that were prospectively found to have the sought activity, which evidences the complementarity of the models.

### 3.5 Comparison to the Results from Applying AI for Drug Repurposing to Other Pathogens

By prospectively examining a library of 6747 compounds, this study identified 9 drugs with potent *S. pneumoniae* activity for the mere cost of purchasing and assaying 11 compounds. Among them, thiostrepton was found to be a highly potent antibiotic (IC_50_ = 60.1 pM), effective against multidrug‐resistant pneumococci, despite its low similarity to any molecule in the training dataset. Table 4 summarizes three additional studies that, to our knowledge, have employed AI for drug repurposing to identify antibiotics. There are many factors affecting the maximum potency of the identified compounds (e.g., different screening libraries, assay methodologies, targeted pathogens, and activity thresholds). Nonetheless, the potency of thiostrepton is about four orders of magnitude higher than the lead compounds identified in these other studies.

**TABLE 4:** Prospective applications of AI models predicting whole‐cell antibacterial activity for drug repositioning. The prospective performance of these AI models cannot be quantitatively compared as they employed different screening libraries, assays, human pathogens and activity thresholds. However, these results collectively show how useful this approach to fight AMR is. NB: HR and MT stand for hit rate and multi‐task, respectively.

Study	Year	Target	AI model	Screened molecules	HR (threshold)	Best potency
Stokes et al. [32]	2020	*E. coli*	D‐MPNN	6680 drugs	51.5% (50 µM)	2 µg/mL MIC
Liu et al. [33]	2023	*A. baumannii*	D‐MPNN ensemble	6680 drugs	3.75% (50 µM)	2 µg/mL MIC
Olayo‐Alarcon et al. [34]	2025	*S. aureus*	MT MolE‐XGBoost	2320 drugs	3.3% (8 µg/mL)	8 µg/mL MIC
This study	2026	*S. pneumoniae*	MoLFormer ensemble, D‐MPNN ensemble and RF	6747 drugs	81.8% (0.4 µg/mL)	10^−4^ µg/mL (0.060 nM) IC_50_

Furthermore, studies in Table 4 collectively demonstrate the benefits of AI‐driven approaches to substantially reduce both the time and cost associated with HTS against the same pathogen when some data is already available for training. For example, in contrast with the prospective results presented here for the same pathogen, only 7 compounds of the 1200 screened drugs in an HTS were retained as strong actives for *S. pneumoniae* D39 (this represents a hit rate of about 0.6%). The best hit, vanoxerine, had a weak MIC of 26 µg/mL [43].

### 3.6 Ablations

To examine the impact of some of the modeling choices made in our pipeline, we also carried out two retrospective ablations. First, we re‐trained all models without presumed negatives and test their ability to rank the 11 prospectively screened molecules by their predicted MIC (Table S6). MoLFormer and RF both had reduced correlation when trained without the presumed negatives, whilst D‐MPNN showed a very slight increase, indicating for most models the inclusion of presumed MRSA negatives improved their ability to rank prospective compounds. Second, we carried out an analysis of models trained under identical train‐test setups. RF and D‐MPNN were re‐trained to match the strategy used when training MoLFormer, in which an ensemble of 5 models was trained, each with one cluster left out. We then generated predictions for the molecules on the drug repurposing hub by taking the median of each ensemble prediction and comparing models under this unified setup in Figure S1 and Table S7. Under the unified setup, the results followed the same pattern as for the original models: MoLFormer ranks on the drug repurposing hub were the most unique, as shown in Figure S1. MoLFormer was also still the most effective model at ranking the prospectively screened molecules, as shown in Table S7, with D‐MPNN and RF rank correlation increasing slightly, but remaining low, overall indicating our varied train‐test split strategies for each model had minimal impact on results.

## 4 Conclusions

This analysis demonstrates the potential of diverse AI models to rapidly screen chemical libraries to identify molecules with anti‐bacterial properties. This approach enables a more rational selection of antibiotics for treatment of pneumococcal diseases. The method most correlated with prospective molecules IC_50_ was an ensemble of transformer‐based MoLFormer models, each incorporating the information from 111 million unlabeled chemical structures during its self‐supervised pretraining step. Such large‐scale representation learning seems to make MoLFormer well suited for predicting the activities of drugs with chemical structures dissimilar to those in the labeled training set. However, the MoLFormer ensemble was not used in isolation, instead it was complemented by ensembles of graph neural networks (D‐MPNN ensemble) and ensembles of CART models (RF). It is this integration of diverse AI models that achieved the very high 82% rate. These results highlight both the promise of large pre‐trained transformers in antibiotic discovery and the value of blending them with complementary approaches, warranting further prospective virtual screening studies that leverage multiple diverse AI models.

The set of molecules selected for validation in vitro highlighted two benefits of a rational approach to phenotypic drug repurposing. When evaluating different beta‐lactam molecules, it identified the highly‐effective cephalosporin‐antibiotic ceftiofur. This is currently only used in veterinary medicine [66, 76] but was more effective against tested penicillin non‐susceptible pneumococci than cefotaxime, commonly used as a broad‐spectrum cephalosporin‐antibiotic in humans [77]. This is consistent with pneumococcal resistance to these antibiotics being driven by the modification of cell wall‐synthesizing penicillin‐binding proteins [78]. The changes to these enzymes are shaped by selection to minimize their affinity for the beta‐lactams to which they are exposed during carriage. Therefore, rarely‐used cephalosporins such as ceftiofur, which differ structurally from those that shape penicillin‐binding protein evolution, may better retain their species‐wide efficacy against pneumococci.

Such quantitative improvements in the effectiveness of cephalosporins can be important when presented with cases of the most severe pneumococcal infections, for which they are a standard treatment. The relatively poor penetration of drugs into the cerebrospinal fluid limits their effectiveness against pneumococci that have passed the blood‐brain barrier [79]. This is particularly problematic in situations such as rising cases of penicillin non‐susceptible pneumococcal meningitis, as observed for serotype 24F in France after the introduction of the 13‐valent pneumococcal polysaccharide conjugate vaccine [80]. Further detailed characterization of the optimal cephalosporin for targeting circulating resistant pneumococci may avoid treatment failures for invasive infections caused by resistant pneumococci.

The structurally distinct molecule thiostrepton was similarly highly effective against both penicillin susceptible and non‐susceptible pneumococci. Like ceftiofur, this drug is currently only used in veterinary medicine [69]. However, its poor solubility in water limits its use to treating non‐invasive infections, such as otitis, as a component of an ointment. Such mixtures typically include other antibiotics, as thiostrepton differs from third‐generation cephalosporins in having a narrower spectrum of activity, being ineffective against many gram‐negative bacteria. However, these properties are beneficial when considering the treatment of common non‐invasive pneumococcal infections of the eyes, ears and sinuses. The current treatment of these diseases with oral penicillins exposes the rest of the microbiotia to these antibiotics, driving a major contribution to bystander selection for beta‐lactam resistance in pathogens such as *S. aureus* and *E. coli* [81]. Yet if molecular diagnostics could distinguish pneumococcal non‐invasive infections from those caused by common gram–negative nasopharyngeal commensals (e.g. *Haemophilus influenzae* or *Moraxella catarrhalis*), then thiostrepton could be a potent treatment for the former infections with the additional advantage of not causing any wider bystander selection across an individual's microbiota. Furthermore, given its efficacy against pneumococci resistant to chloramphenicol, tetracycline and macrolides, there is no evidence of treatment with thiostrepton driving cross‐resistance against other classes of antibiotic that can be used systemically. This therapeutic strategy could reduce the prevalence of resistance across multiple opportunistic bacterial pathogens.

Consideration of the spectrum of antibiotics has become part of the drive for better stewardship, using measures such as the WHO AWaRe system [82] and Antibiotic Spectrum Index [83], to guide the use of antibiotics that are more effective against the causative pathogen, while minimizing bystander selection against the broader microbiota. This is intended to overcome the current tendency for prescribing practices for common bacterial infections to be driven by convention, rather than rational drug selection. This study highlights how AI methods can be used not only for discovery of novel antimicrobial lead compounds, but also for short‐term rational selection of treatments from existing drug repurposing libraries. Therefore, AI‐driven quantitative improvements in the effectiveness of systemic antibiotics, and use of narrow‐spectrum topical antibiotics against common infections, have the potential to contribute to the challenge of AMR, while such methods also enable the longer‐term development of novel classes of antimicrobial.

## Author Contributions

P.J.B. conceived the study. P.J.B. and N.J.C. supervised the research. M.D.A. and J.F. wrote the code and carried out all the numerical experiments. N.J.C. helped to design the filters for dataset compilation. P.J.B. and M.D.A. prospectively selected the drugs to test in vitro using the computational model predictions. M.J.K., L.H. and N.J.C. performed and analyzed the microbiological experiments. M.D.A., J.F., S.H.H., N.J.C. and P.J.B. wrote the manuscript.

## Funding

We would like to acknowledge Qianrong Guo for help with discussion over code and algorithms and Imperial College London for providing the employed HPC resources. P.J.B. thankfully acknowledges the Royal Society and the Wolfson Foundation for a Royal Society Wolfson Fellowship (grant RSWF∖R1∖221005). J.F. is supported by UK Research and Innovation (UKRI AI Centre for Doctoral Training in Digital Healthcare grant EP/Y030974/1). M.J.K., L.H. and N.J.C. were supported by a Sir Henry Dale fellowship jointly funded by Wellcome and the Royal Society (grant 104169/Z/14/A) and by the UK Medical Research Council and Department for International Development (grants MR/R015600/1 and MR/T016434/1).

## Conflicts of Interest

The authors declare no conflicts of interest.

## Supporting information




**Supporting File**: advs76959‐sup‐0001‐SuppMat.docx.

## Data Availability

Training and repurposing data are available alongside Python code and pretrained models at: https://github.com/fitchy123/pneumococcusVS_PV.
