# Supplementary material for: Artificial Intelligence‐Guided Phenotypic Drug Repurposing Against Streptococcus pneumoniae
Source: Adv Sci (Weinh). 2026 Sep 16:e76959. Online ahead of print. doi: 10.1002/advs.76959 (PMC13579117; doi:10.1002/advs.76959)
Supplement: Supplementary file 1 [file ADVS-9999-e76959-s001.docx]

**Supplementary**

**Table S1: Dataset cluster composition and count**

| **Cluster ID** | **Positive Proportion (%)** | **Count** | **Total Positives** |
| --- | --- | --- | --- |
| 0 | 0.40% | 3,230 | 13 |
| 1 | 0.04% | 2,490 | 1 |
| 2 | 10.69% | 1833 | 196 |
| 3 | 2.29% | 2,924 | 67 |
| 4 | 0.15% | 1,993 | 3 |
| 5 | 0.30% | 2,003 | 6 |
| 6 | 37.27% | 1,524 | 568 |
| 7 | 0.20% | 1,482 | 3 |
| 8 | 0.00% | 627 | 0 |
| 9 | 0.00% | 1,572 | 0 |
| 10 | 0.06% | 1,730 | 1 |
| 11 | 56.71% | 1,363 | 773 |
| 12 | 0.53% | 753 | 4 |
| 13 | 0.19% | 1,062 | 2 |
| 14 | 3.94% | 1,676 | 66 |
| 15 | 0.00% | 1,043 | 0 |
| 16 | 0.00% | 552 | 0 |
| 17 | 74.73% | 182 | 136 |
| 18 | 0.00% | 522 | 0 |
| 19 | 0.00% | 1,208 | 0 |
| 20 | 0.00% | 509 | 0 |
| 21 | 0.00% | 2,008 | 0 |
| 22 | 0.00% | 1,077 | 0 |
| 23 | 0.00% | 968 | 0 |
| 24 | 0.09% | 1,132 | 1 |
| 25 | 1.01% | 889 | 9 |

**Table S2: Grid search hyperparameter tuning ranges and final selected values for RF**

| **Hyperparameters** | **Choices** | **Selected Value** |
| --- | --- | --- |
| max_depth | None, 16, 256 | None |
| min_samples_leaf | 2, 4 | 4 |
| criterion | ‘gini’, ‘entropy’ | ‘entropy’ |
| class_weight | ‘balanced’, None | ‘balanced’ |

**Table S3: Grid search hyperparameter tuning ranges and final selected values for MoLFormer**

| Hyperparameters | **Choices** | **Selected Value** |
| --- | --- | --- |
| FFN dropout rate | 0.2, 0.4 | 0.4 |
| FFN n layers | 2, 4, 6 | 6 |
| FFN hidden_dim | 768, 1536 | 1536 |

**Table S4. Training hyperparameters and hardware for the D-MPNN deep learning models**

| **Parameter** | **D-MPNN** |
| --- | --- |
| Batch Size | 128 |
| Learning Rate | 1×10⁻³ |
| Optimizer | Adam |
| Epochs | 10 |
| LR Scheduler | None |
| Early Stopping | None |
| GPU | NVIDIA RTX6000 |

**Table S5. Training hyperparameters and hardware for the MoLFormer deep learning models**

| **Parameter** | **MolFormer** |
| --- | --- |
| Batch Size | 256 |
| Learning Rate | 1×10^-4^ |
| Optimizer | AdamW (weight_decay=0.1) |
| Epochs | 5 |
| LR Scheduler | None |
| Early Stopping | None |
| GPU | NVIDIA RTX6000 |

**Table S6. Comparison of Spearman rank correlation coefficients between predicted compound ranks and experimental IC_50_ ranks for the prospective screening set (n = 11) under the original training regime and an ablation in which presumed MRSA negatives are excluded.** Removal of the presumed-negative decoys weakens the rank correlation for the MoLFormer, consensus and RF, while D-MPNN shows a very slight increase.

| **Model** | **Original Rank Correlation (Significance)** | **Ablation Rank Correlation (Significance)** | **ΔCorrelation (Ablation − Original)** |
| --- | --- | --- | --- |
| Consensus of MoLFormer and D-MPNN ensembles Rank | 0.74 (0.0098) | 0.43 (0.19) | −0.31 |
| MoLFormer | 0.75 (0.0073) | 0.46 (0.15) | −0.29 |
| D-MPNN | 0.15 (0.65) | 0.16 (0.63) | +0.014 |
| Random Forest | 0.16 (0.63) | 0.0091 (0.98) | −0.15 |

**Table S7. Spearman rank correlation between predicted compound ranks and experimental IC_50_ ranks for the prospective screening set (n = 11) under the unified standard-split training regime, where each model is an ensemble of 5 with clusters 2, 3, 12, 14 or 25 held out and the median ensemble prediction is used.**

| **Model** | **Rank Correlation** | **Significance (p-value)** |
| --- | --- | --- |
| MoLFormer | 0.75 | 0.0073 |
| D-MPNN | 0.18 | 0.59 |
| Random Forest | 0.24 | 0.48 |
| Consensus of MoLFormer and D-MPNN ensembles | 0.72 | 0.013 |
|  |  |  |

**Figure S1:** **Analysis of the diversity of different AI model outputs on the drug repurposing hub under a standard split where each model is an ensemble of 5 with clusters 2,3,12,14 or 25 held out, median prediction of the ensemble is used.** **a)** Percentage overlap of molecules in the top-n-quantile of ranks for each model. Shaded regions are 95% confidence interval calculated with a normal approximation for a binomial proportion. **b)** Plot of the proportion of molecules unique to a model, defined as the fraction of molecules in the top n% of a model that do not appear in the top n% of any other model. Shaded regions were calculated the same way is in panel (a).
